# Healthcare information technology in medical education – a forgotten focus

**DOI:** 10.3402/meo.v20.30191

**Published:** 2015-12-15

**Authors:** Hammad H. Malik, Ibtesham T. Hossain

**Affiliations:** School of Medicine, Imperial College London, London, UK

Information technology has been pivotal in transforming the face of modern health care. Healthcare information technology (HCIT) is a term used to describe the use of computers in a clinical setting (1). HCIT's benefits include, but are not limited to: written electronic patient records, increased delivery of guideline-based care, easier access to investigation results, reduction of medical errors, and decreased rates of administration of potentially inappropriate care (2). HCIT generally forms a part of everyday practice across all specialties, with computer systems now heavily integrated into many clinical encounters. Recognising the importance of information technology at large, the General Medical Council (GMC) advocates that medical graduates should be able to ‘make effective use of computers and other information systems, including storing and retrieving information’ (3).

A brief analysis of current prospectuses, course curriculums, and content maps available on the worldwide web for UK medical schools fails to return any overt emphasis on HCIT. Although most of these documents quite-rightly focus on preclinical and clinical themes to form the basis of their content, this suggests that HCIT teaching is not a priority in undergraduate medical education. It may be argued that there is in fact a complete lack of teaching on the theme in some institutions. This is a worrying prospect, as interactions involving HCIT may form the majority of clinical encounters for modern medical graduates.

Given that the utilization of HCIT is only likely to increase, this heralds its introduction through formal pedagogy within undergraduate medical education. Through outlining and teaching core competencies in the subject, students can be empowered to uphold effective communication skills whilst engaging with computerised devices. This may also help them consider the financial, ethical, and legal vulnerabilities pertaining to health informatics. Ultimately, this would help produce well-rounded physicians who are literate in both medical sciences and HCIT, enabling them to utilise the full benefits of the technology to improve the quality of the care they deliver.

A pioneering curriculum designed to deliver effective HCIT teaching has been developed and implemented in the United States, with key objectives including training students in electronic health records (EHR)–related skills and empowering patient-centred interviewing whilst incorporating the aforementioned EHR skills (4). Through integrating these key objectives into a spiral curriculum and revisiting content over the entire medical course at the Alpert Medical School of Brown University (USA), the authors demonstrated deep learning amongst medical students. Furthermore, students appreciated the importance of computer use, upheld engagement of patients with their healthcare narrative, and fostered empathic communication and care.

In conclusion, we highlight a need for modern medical curriculums to incorporate purposefully designed objectives relating to HCIT. Medical schools are in the best position to determine the ideal manner of ensuring that future doctors develop the core competencies required to use the technology. Once cemented in medical education, tomorrow's doctors will be able to effectively balance HCIT with other clinical demands, in order to practice patient-centred care.

*Hammad H. Malik* School of Medicine Imperial College London London, UK Email: hhm10@ic.ac.uk*Ibtesham T. Hossain* School of Medicine Imperial College London London, UK
